# Susceptibility to Anthrax Lethal Toxin-Induced Rat Death Is Controlled by a Single Chromosome 10 Locus That Includes *rNlrp1*

**DOI:** 10.1371/journal.ppat.1000906

**Published:** 2010-05-20

**Authors:** Zachary L. Newman, Morton P. Printz, Shihui Liu, Devorah Crown, Laura Breen, Sharmina Miller-Randolph, Pamela Flodman, Stephen H. Leppla, Mahtab Moayeri

**Affiliations:** 1 National Institute of Allergy and Infectious Diseases, National Institutes of Health, Bethesda, Maryland, United States of America; 2 Department of Pharmacology, University of California-San Diego, La Jolla, California, United States of America; 3 Department of Pediatrics, University of California, Irvine, Irvine, California, United States of America; The University of Texas-Houston Medical School, United States of America

## Abstract

Anthrax lethal toxin (LT) is a bipartite protease-containing toxin and a key virulence determinant of *Bacillus anthracis*. In mice, LT causes the rapid lysis of macrophages isolated from certain inbred strains, but the correlation between murine macrophage sensitivity and mouse strain susceptibility to toxin challenge is poor. In rats, LT induces a rapid death in as little as 37 minutes through unknown mechanisms. We used a recombinant inbred (RI) rat panel of 19 strains generated from LT-sensitive and LT-resistant progenitors to map LT sensitivity in rats to a locus on chromosome 10 that includes the inflammasome NOD-like receptor (NLR) sensor, *Nlrp1*. This gene is the closest rat homolog of mouse *Nlrp1b*, which was previously shown to control murine macrophage sensitivity to LT. An absolute correlation between *in vitro* macrophage sensitivity to LT-induced lysis and animal susceptibility to the toxin was found for the 19 RI strains and 12 additional rat strains. Sequencing *Nlrp1* from these strains identified five polymorphic alleles. Polymorphisms within the N-terminal 100 amino acids of the Nlrp1 protein were perfectly correlated with LT sensitivity. These data suggest that toxin-mediated lethality in rats as well as macrophage sensitivity in this animal model are controlled by a single locus on chromosome 10 that is likely to be the inflammasome NLR sensor, *Nlrp1*.

## Author Summary

Inflammasomes are multiprotein cytoplasmic complexes that respond to a variety of danger signals by activating the host innate immune response. The sensor components of these complexes are NLR (NOD-like receptor) proteins. In this report, a recombinant inbred rat strain collection was used to genetically map anthrax lethal toxin (LT) susceptibility to a limited region of chromosome 10 containing one such sensor, *Nlrp1*. Similar to its mouse ortholog, *Nlrp1b*, which controls murine macrophage sensitivity to this toxin, the locus containing rat *Nlrp1* was shown to control macrophage sensitivity to anthrax LT. However, unlike the situation in mice, where multiple genetic loci influence animal susceptibility to LT, the single chromosome 10 locus alone appears to control the rapid anthrax LT-induced death, which can occur in as little as 37 minutes. Sequencing of *Nlrp1* from 12 rat strains identified polymorphisms which correlated perfectly with animal sensitivity to toxin. These polymorphisms were within the N-terminal 100-amino acid portion of Nlrp1, in an area of unknown function, which suggests that the N-terminus of rodent Nlrp1 could be an important functional domain.

## Introduction

Anthrax lethal toxin (LT), a major virulence factor of *Bacillus anthracis*, is composed of two proteins, lethal factor (LF) and protective antigen (PA). PA binds to cellular receptors and facilitates LF entry into the cytosol (for review see [1]). LF is a protease which cleaves and inactivates members of the mitogen-activated protein kinase kinase (MAPKK or MEK) family, resulting in proliferation arrest in most cell types and a unique, rapid (<90 min), caspase-1 dependent lysis of mouse macrophages from certain inbred strains through poorly characterized mechanisms (for review see [2]).

In mouse macrophages, sensitivity to LT-mediated lysis is a dominant trait that maps to the highly polymorphic *Nlrp1b* (*Nalp1b*) gene on chromosome 11 [3]. Mouse *Nlrp1b* (*mNlrp1b*) has five alleles that correlate with LT sensitivity or resistance in macrophages, and it is one of three tandem *Nlrp1* paralogs on chromosome 11 [3]. mNlrp1b, the paralog controlling LT sensitivity, is a NOD-like receptor (NLR) which, when activated, leads to assembly of the inflammasome, a multiprotein complex responsible for the activation of caspase-1 [4]. The mNlrp1b inflammasome-mediated activation of caspase-1 is necessary for murine macrophage cell death in response to LT [3], [5]–[7]. Furthermore, expression of mNlrp1b from LT-sensitive macrophages together with caspase-1 is sufficient to render other cell types sensitive to the effects of LT [8].

Unlike human Nlrp1 (hNlrp1), mNlrp1b (despite the acronym representing NLR family, pyrin domain containing 1b) lacks an N-terminal pyrin domain. The pyrin domain is required for hNlrp1 binding to the inflammasome adaptor protein ASC, and mNlrp1b is not believed to interact with this adaptor [9]. However, mNlrp1b does have the NACHT (nucleotide oligomerization), LRR (leucine-rich repeat), and CARD (caspase recruitment) domains commonly found in NLR proteins (for recent reviews see: [10], [11]). It is unclear how polymorphisms in the mNlrp1b protein result in such striking variation in the ability of LT to activate caspase-1 (and subsequently induce cell death) in murine macrophages.

While the mNlrp1b inflammasome requirement for murine macrophage death in response to LT is well established, it is unclear if this inflammasome is involved in LT-mediated death of animals. LT injection into rodents induces an atypical vascular collapse, replicating the shock state associated with anthrax disease [12]–[14]. Susceptibility in mice, however, is controlled by multiple loci [15], and macrophage sensitivity does not control animal susceptibility to LT [16]. Furthermore, factors such as adrenal function can also modulate LT toxicity in mice [17]. Thus, the molecular basis for the death induced by LT in mice is currently unknown.

The rapid LT-mediated death of the Fischer rat [18] can occur in as little as 37 minutes through a unique vascular shock [19], [20]. In rats, left ventricular failure accompanied by a rapid accumulation of pleural fluid (a hallmark of anthrax disease) is typically associated with LT-mediated death [12], [21], [22]. In contrast, LT-induced murine death occurs by vascular collapse over a longer period of days [13], [15]. Early targeting of cardiac function by LT has also recently been demonstrated in mice [23]. The role of MEK cleavage and/or inflammasome components in vascular collapse induced by LT in rodents is also currently unknown. Thus, studies of determinant molecular pathways would be greatly assisted if genomic targets controlling susceptibility were identified.

Strain-specific variations in macrophage and animal sensitivity to LT were previously noted for four rat strains [24]. Toxicity testing of first filial (F1) progeny from crosses of LT-sensitive (Brown Norway and F344) and resistant (Lewis and Wistar Kyoto) strains led to the conclusion that toxin sensitivity exhibited a dominant mode of inheritance [24]. Further, the authors concluded that the inter-cross results were consistent with LT sensitivity in rats being determined by a single, dominant gene [24].

In the current report, we used the HXB/BXH recombinant inbred (RI) rat collection, developed by two gender-reciprocal matings of the Wistar Kyoto-related strain, the SHR/Ola rat (an LT-resistant rat) with a Brown Norway congenic (BN-Lx, an LT-sensitive rat) [25], [26] as an ideal genetically-derived animal model to map LT sensitivity of rats. We report that susceptibility of rats to anthrax LT maps to a single locus on chromosome 10 that contains the *Nlrp1* (*rNlrp1*) gene. Furthermore, LT sensitivity of a large number of rat strains was found to perfectly correlate with their macrophage sensitivity to toxin. Sequence analysis of *rNlrp1* from twelve rat strains identified specific variations within a limited 100-aa N-terminal region of rNlrp1 that correlate perfectly with LT sensitivity. Taken together, these data suggest that a single locus on chromosome 10, likely *rNlrp1*, controls both rat macrophage sensitivity to anthrax LT as well as rat death in response to this toxin.

## Results

### Susceptibility to LT in the rat correlates with macrophage sensitivity to toxin

**Figure 1:**
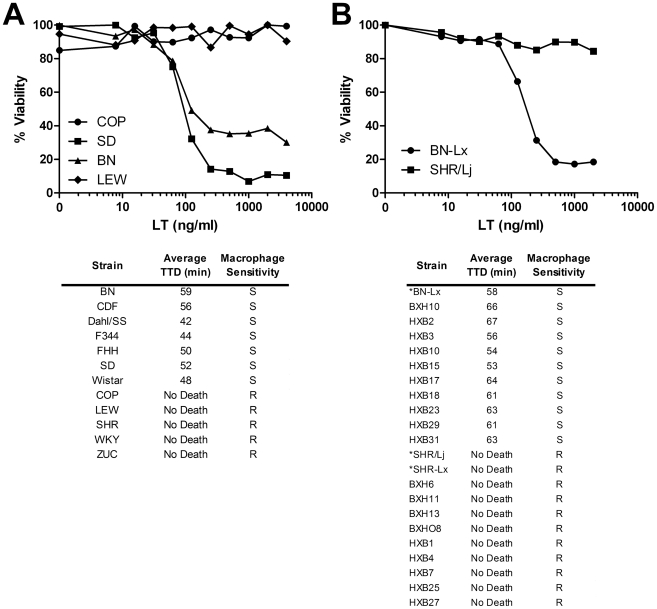
LT sensitivity in rat strains correlates perfectly with macrophage sensitivity. (A, B) Top panels show representative cytotoxicity assays for rat macrophages treated with toxin. BMDMs were treated with various concentrations of LT and cell viability was assessed after 3 h by MTT assay as described in Materials and Methods. Lower panels show average rat times to death (TTD) after LT treatment (100 µg, IV, n = 3 or n = 4/strain), and the macrophage LT sensitivity phenotypes (S  = sensitive, R = resistant). (*)s indicate progenitor strains and a related congenic strain.

Twelve inbred rat strains and their bone marrow-derived macrophages (BMDMs) were tested for sensitivity to anthrax LT (Figure 1A). Both the rats and their corresponding BMDMs exhibited a qualitative dichotomous phenotype. Thus, macrophages were either sensitive or resistant to toxin, and rats showed an identical pattern, either succumbing within 60 minutes or remaining completely resistant to systemic toxin treatment (Figure 1A). For all the inbred rat strains, sensitivity of BMDMs was predictive of animal susceptibility to toxin. This result is notably different from what was previously observed in a comparison of mouse strains, where correlation between mouse strain susceptibilities to LT and their macrophage sensitivities was not found [15]. We and others have proposed that multiple genetic loci control LT susceptibility in mice [15], [27].

### Recombinant inbred rat screen identifies the LT susceptibility locus in rats

The absence of intermediate sensitivities in the rats supported the possibility that LT sensitivity in rats is controlled by a single gene [24]. This fact suggested that a recombinant inbred (RI) rat strain panel derived from LT-sensitive and LT-resistant progenitors could be used to map the LT susceptibility locus. RI strains allow linking of allelic variation at specific chromosomal loci to particular phenotypes. The widely used HXB/BXH RI rat collection was developed by two gender-reciprocal matings of the hypertensive SHR/Ola rat (an LT-resistant rat; “H” alleles) with a Brown Norway (BN) congenic expressing polydactylyl luxate syndrome (BN-Lx, an LT-sensitive rat; “B” alleles) [25], [26]. This RI panel has been successfully used for identification of quantitative trait loci that control a range of phenotypes, including cardiovascular function, insulin resistance and multiple behavioral traits (for review see [26]). We tested nineteen HXB/BXH RI rat strains and their macrophages for sensitivity to toxin. Ten of nineteen strains were sensitive, and once again the LT sensitivity of their isolated macrophages correlated perfectly with animal susceptibility (Figure 1B). Differences in PA receptor function were ruled out as progenitor strains had similar sensitivity to an LF-*Pseudomonas* exotoxin A fusion protein (FP59), which requires PA for cell entry but induces lethality by inhibition of protein synthesis (data not shown).

**Figure 2:**
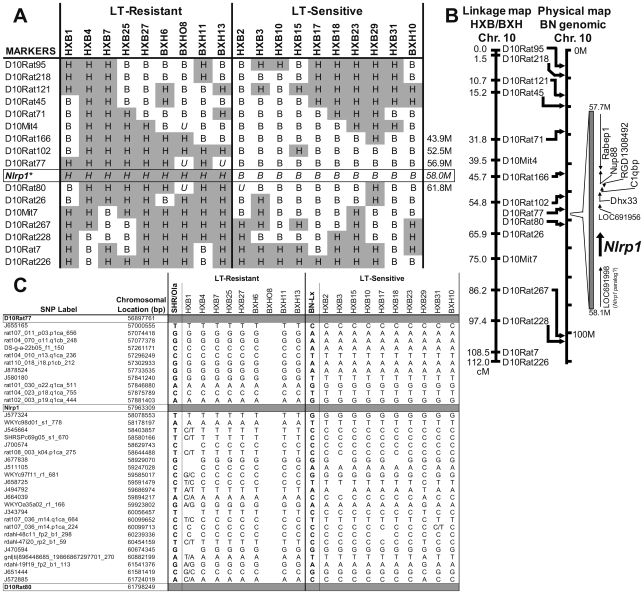
Mapping of the LT sensitivity locus to chromosome 10. (A) Strain distribution patterns at several markers on chromosome 10 (a complete collection based on published data [28] for all chromosomes is found in Figure S1). Genotypes are SHR-like (H; gray) or BN-Lx-like (B; white). *U* indicates unknown genotypes. (*) indicates predicted genotypes. (B) Linkage map for markers on chromosome 10. Markers with known locations in the genomic data for chromosome 10 of the Brown Norway rat (BN/SsNMcw; RGSC 3.4) are mapped to their approximate physical locations on the chromosome (indicated with arrows). Expansion indicates the region from 57.7 M to 58.1 M of chromosome 10 and selected open reading frames. (C) SNP data between markers D10Rat77 and D10Rat80 (flanking *rNlrp1*) for all RI strains (except BXHO8) and their progenitors. SNP heterozygosity is indicated by dual base designation.

Analyses of LT sensitivity phenotypes in the context of published marker data for all chromosomes of the RI rat panel [28] pointed to the existence of a single sensitivity locus on chromosome 10, with the strongest linkage being to marker D10Rat102 (52.5 M), where marker genotypes matched sensitivity and resistance to LT in all but two rat strains (P = 0.001) (Figure 2A and Figure S1). An abridged set of markers (O. Seda and L. Sedova, unpublished) that were mapped to the initial marker set for agreement (P. Flodman et al., unpublished) indicated that the genotype at another marker (D10Rat77, 56.9 M) was fully consistent with the LT phenotype for all genotyped RI strains (P = 0.00005; Figure 2A). We noted that *rNlrp1* (58.0 M), the ortholog for the murine LT macrophage sensitivity locus, lies within 1.1 M of D10Rat77 (Figure 2B). Analysis of SNP data in the region between marker D10Rat77 and marker D10Rat80 (also from the unpublished abridged data set) showed a perfect correlation between genotype and LT phenotype in all rat strains for the region surrounding *rNlrp1* (P = 0.00001; Figure 2C). The boundaries of the LT susceptibility locus were determined by SNP analyses of all RI strains to lie between SNP Cpn_10055303964 at 55.3 M and WKYc98d01_s1_778 at 58.2 M (http://gscan.well.ox.ac.uk/gsBleadingEdge/rat.snp.selector.cgi). Analyses of SNPs within this locus comparing RI progenitor strains to several of the previously characterized inbred strains (COP, LEW, WKY, Dahl/SS, and F344) found only 7 individual SNPs that perfectly correlated with sensitivity among these LT sensitive and LT resistant strains. Three of these SNPs lie very close to *rNlrp1* (rat101_030_o22.q1ca_511 at 57.8 M, rat102_003_p19.q1ca_444 at 57.9 M, and J577324 at 58.1 M), further supporting *rNlrp1* as the leading candidate sensitivity locus among a small number of candidate genes. In view of the prior demonstration that *mNlrp1b* controls mouse macrophage sensitivity to LT, it was not surprising that the mapping data identified a locus containing *rNlrp1* as determining LT sensitivity in rat macrophages. However, a single locus control of animal death was not anticipated.

BLAST searches (http://blast.ncbi.nlm.nih.gov/Blast.cgi) using the predicted BN *rNlrp1* (allele 1, see later sections) sequence also identified a potential *rNlrp1* paralog (GenBank accession: XM_001080760 “similar to NACHT, leucine rich repeat and PYD containing 1” at LOC691998 in the Rat Genome Sequencing Consortium (RGSC) v3.4, or alternatively, XM_001080056 at LOC687768 in the Celera assembly) located immediately adjacent to *rNlrp1* with a predicted protein sequence that has 76% aa identity with the BN rNlrp1 sequence. RT-PCR analyses utilizing intraexonic and intron-spanning primers specific to this paralog showed that it exists in all strains except Copenhagen (COP) but is not expressed in macrophages and thus it is unlikely that this paralog is involved in macrophage toxicity (Figure S2).

### rNlrp1 N-terminal sequence in rats correlates with LT sensitivity

**Figure 3:**
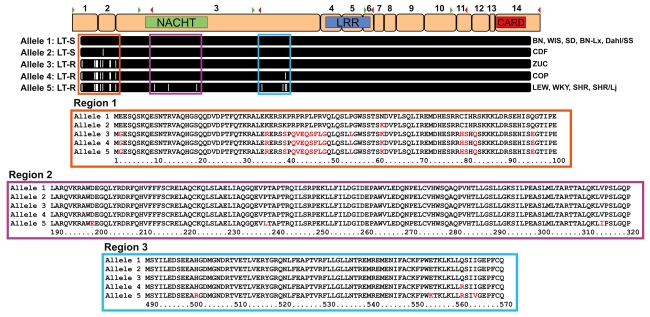
Allelic variations in *rNlrp1* correlate with LT sensitivity. Proposed exon structure of the mature mRNA for *rNlrp1* is shown at the top. Exons are numbered above and locations of forward (green arrowhead) and reverse (red arrowhead) primers for the primary sequencing reactions are indicated. Approximate domain locations are shown for NACHT (green), LRR (blue), and CARD (red). Amino acid alignments for the five alleles of rNlrp1 are aligned to the exon structure. White hashes are indicative of amino acid changes relative to protein encoded by allele 1. Expansions of the three regions of interest are also shown with alignments of the five alleles. Red letters identify residues that differ from those in the protein encoded by allele 1.

The perfect correlation of macrophage sensitivity and animal susceptibility to toxin, along with the established role of *mNlrp1b* in controlling murine macrophage sensitivity suggested that *rNlrp1* was the best candidate for control of sensitivity. We next sequenced *rNlrp1* cDNA from twelve rat strains in order to identify sensitivity-correlated variations. BMDMs isolated from ten strains analyzed previously (Figure 1) as well as the progenitor strains for the RI panel (Figure 2) were used as sources of mRNA for sequencing. The sequencing identified a 3657-bp coding region corresponding to a 1219-aa rNlrp1 protein. By aligning the cDNA sequences to the BN genomic sequence data we determined that the *rNlrp1* mRNA is formed through splicing of 14 exons, arranged like those of *mNlrp1b* [3]. Conserved Domain Database (CDD) searches indicated that rNlrp1 contains the same functional NACHT (nucleotide oligomerization; pfam05729), LRR (leucine-rich repeat; cd00116), and CARD (caspase recruitment; pfam00619) domains as mNlrp1b and hNlrp1 (Figure 3). rNlrp1 is similar to mNlrp1b in lacking a pyrin domain. Sequence alignments showed there to be two sensitivity-associated (“sensitive”) alleles (1 and 2) and three resistance-associated (“resistant”) alleles (3,4,5) among the studied strains (Figure 3 and Figure S3). The differences within these two groups turn out to be minor. The protein encoded by the second sensitive allele, allele 2, differs from allele 1 at a single amino acid (Asn^61^ to Lys^61^), but this substitution is also found in resistant alleles 3–5. More interestingly, alleles 3 and 4 both contain sequences corresponding to the N-terminal region of resistant allele 5 and several polymorphisms associated with the C-terminus of sensitive allele 1. Thus, the predicted proteins in these two rats combine a few elements of both a resistant and a sensitive rNlrp1, making it unlikely that the NACHT, LRR and CARD domains determine LT susceptibility. A single difference between resistant alleles 3 and 4 was found, where a substitution results in a Gln to Arg change (Arg^561^). Allele 5, however, is similar to allele 4 and contains this Arg^561^ residue, indicating that this residue is also unlikely to be associated with resistance to LT. Thus, we conclude that all the polymorphisms that correlate perfectly with LT sensitivity lie in the 100-aa N-terminus of rNlrp1. Unfortunately, no information is available about the function of this region in rodent Nlrp1 proteins. There is no homologous region in hNlrp1, which instead harbors an N-terminal pyrin domain (absent from rodent Nlrp1 proteins) [10].

## Discussion

In the work reported here, a RI rat panel was used to identify the LT susceptibility locus for both rats and their macrophages. Analyses of LT sensitivity in several rat strains as well as the RI panel identified a complete correlation between macrophage and rat sensitivity to the toxin. This locus, on chromosome 10, contains *rNlrp1*, which is the homolog for the mouse *mNlrp1b* gene, previously proven to be critical for determining murine macrophage susceptibility to LT. Sequence analyses of *rNlrp1* in primary macrophages from twelve rat strains identified five polymorphic alleles. Surprisingly, the few polymorphisms that correlated with LT macrophage and animal sensitivity in rats were located within the first 100 aa of rNlrp1, in an area of undefined function, and not within the previously described Nlrp1 functional domains (NACHT, LRR and CARD).

The mapping data strongly suggests (with a P = 0.000001) that this *rNlrp1-*containing region of chromosome 10 is the LT sensitivity locus for both rats and their macrophages. Among the approximately 250 microsatellite markers previously characterized for this RI set [28] and new previously unpublished markers sets, we found that marker D10Rat77 on chromosome 10 had an absolute genotypic correlation with the LT sensitivity phenotype. SNP analysis in this region also confirms the marker data and shows perfect correlation for LT susceptibility within the locus containing *rNlrp1* (genome-wide empiric p-value = 0.001). Our mapping data does not rule out the possibility that another gene very closely linked to *rNlrp1* could be mediating LT's effects in the rat. However, two additional lines of evidence argue against this possibility. First, rNlrp1 aa sequence variations between several inbred rat strains unrelated to the RI panel progenitors correspond perfectly with the sensitivity phenotypes. Second, an absolute correlation was found between macrophage sensitivity and animal susceptibility for 34 rat strains. Considering the established role of *mNlrp1b* in control of murine macrophage sensitivity, it is unlikely that a different gene controls macrophage sensitivity in rats. However we cannot exclude the possibility that whole animal susceptibility is controlled by multiple closely-linked genes within the single chromosome 10 locus identified in this study, and that these genes are inherited in a fashion such that polymorphisms associated with sensitivity in *rNlrp1* are also always found in another candidate gene. Historically, a similar issue plagued the identification of *mNlrp1b* as the mouse macrophage sensitivity locus for LT. The sensitivity locus in mice was first identified as the closely linked *Kif1c* gene, which presented almost perfect polymorphism correlations with sensitivity [29]. In the absence of a transgenic rat model providing definitive proof linking the *rNlrp1* gene to rat death, we believe the mapping and sequence data presented here strongly support *rNlrp1* as the most likely determinant of LT sensitivity.

Gene predictions and BLAST searches identified a potential paralog immediately adjacent to *rNlrp1*, but this paralog is not expressed at the mRNA level, at least in macrophages. Similarly, of the three tandem *mNlrp1* paralogs found in mice, only *mNlrp1b* was shown to be expressed in the LT-sensitive 129S1/SvImJ macrophage, and expression of this paralog was sufficient to confer LT sensitivity to resistant mouse macrophages and fibroblasts [3], [8]. However, the other two mouse paralogs are expressed in a number of inbred strains, further complicating analyses of mouse susceptibility. Curiously, phylogenetic analyses indicate that the predicted rNlrp1 paralog sequence is distant from the other rat Nlrp1 sequences and is more similar to the mouse and human Nlrp1 sequences (Figure S4).

The highly polymorphic nature of the *mNlrp1b* alleles has made it difficult to associate specific polymorphisms with the macrophage sensitivity phenotype in mice. Fortunately, there are far fewer differences in *rNlrp1* between sensitive and resistant rat strains. Sequence differences that correlated with phenotypic differences were found only within the extreme N-terminal region of rNlrp1, and not in the domains (NACHT, LRR, and CARD) which have recognized roles in Nlrp1 function. This surprising finding draws attention to the N-terminal domains of rodent Nlrp1 proteins, absent in hNlrp1, which instead contain a pyrin domain at the N-terminus [11]. The pyrin domain in hNlrp1 is required for association with the inflammasome adaptor protein ASC, which is not part of the LT-induced mNlrp1b inflammasome complex [9]. Interestingly, all human macrophages tested to date have been LT-resistant (unpublished observations), a behavior that might relate to the absence of the N-terminal pyrin domain in rodent Nlrp1. However, hNlrp1 polymorphisms are now being identified and associated with a number of human diseases [30]–[32], so it may be necessary to test a larger number of donors to identify any LT-sensitive *hNlrp1* alleles. As the N-terminus in hNlrp1 plays an important role in protein-protein interactions, it is tempting to postulate that the N-terminal 100 aa of rodent Nlrp1 proteins may also interact with other cellular components to modulate function.

The perfect correlation of rat macrophage LT sensitivity to that of the animals might at first suggest that the lysis of macrophages *in vivo* causes the rapid death of LT-injected rats. However, this is unlikely to be the case, for several reasons. Rat macrophages begin to die only 2 h after treatment *in vitro* with saturating toxin doses, whereas the rats may die in as little as 37 min [20]. Initial studies in LT-treated mice were interpreted as showing that death (which occurs only after 2–3 days) resulted from cytokines released following macrophage lysis [33]. However, more extensive later studies showed that mice harboring resistant macrophages also succumb to LT through a vascular collapse that is similar to that in mice with sensitive macrophages [13], and the correlation within mouse strains between the LT sensitivities of isolated macrophages and the animals is poor [13], [15]. Studies with mNlrp1b transgenic mice confirm that macrophage and animal susceptibility to LT are not correlated [16]. Preliminary studies in our laboratory suggest that cell types other than macrophages control the lethal response to LT (data not shown). Consistent with this view, Nlrp1 has recently been demonstrated to play a functional role in a number of cell types, including neuronal cells [34]–[37]. Furthermore, it should be noted that LT-induced death in both rats and mice has recently been associated with early changes in cardiac function [21], [23]. Thus, it is possible that LT targeting of rNlrp1 function in the heart plays a role in the rapid lethality phenotype. A better understanding of the distribution and function of different Nlrp1 isoforms in various cell types is needed to fully understand the mechanisms by which LT may influence Nlrp1 activity, and whether this gene alone is sufficient for control of animal susceptibility to toxin.

In summary, we present data mapping the control of rapid LT-induced rat death to a single chromosome 10 locus. This locus contains the polymorphic *rNlrp1* gene, which is the best candidate for conferring sensitivity to macrophages, and possibly to animals. As such, this is the first suggestion that an inflammasome NLR protein may directly control animal lethality. While both the mechanistic basis for the rapid LT-induced lethality in the rat and direct proof of rNlrp1-mediated rat death require further experimentation, identification of the limited polymorphisms within rNlrp1 that correlate perfectly with LT sensitivity suggest a starting point for analysis of the possible role this protein may play in controlling rapid rat death in response to LT.

## Materials and Methods

### Ethics statement

All animal experiments were performed in strict accordance with guidelines from the NIH and the Animal Welfare Act, under protocols approved by the Animal Care and Use Committee of the National Institute of Allergy and Infectious Diseases, National Institutes of Health.

### Animal studies

PA, LF, and FP59 were purified from *B. anthracis* [38]–[40]. The LF used here is a recombinant protein having an N-terminal sequence beginning HMAGG. Doses and concentrations of LT given for each experiment correspond to that of each toxin component (i.e., 1 µg/ml LT is 1 µg/ml PA +1 µg/ml LF and 100 µg LT is 100 µg PA +100 µg LF). Rats purchased from Charles River Laboratories (Wilmington, MA) were maintained there as either inbred or long-term outbred colonies. Rats strains used included (with strain designations, abbreviations and inbred/outbred status): Brown Norway (BN/Crl; BN; inbred), Fischer CDF (F344/DuCrl; CDF; inbred), SASCO Fischer (F344/NCrl; F344; inbred), Dahl Salt Sensitive (SS/JrHsdMcwiCrl; Dahl/SS; inbred), Lewis (LEW/Crl; LEW; inbred), Wistar (Crl:WI; WIS; outbred), Wistar Kyoto (WKY/NCrl; WKY; inbred), Sprague Dawley (CRL:SD; SD; outbred), Spontaneously Hypertensive Rat (SHR/NCrl; SHR; inbred), Copenhagen (COP/CrCrl; COP; inbred), Zucker-Lean (Crl:ZUC-Leprfa; ZUC; outbred) and Fawn Hooded Hypersensitive (FHH; inbred). The recombinant inbred (RI) rat strain panel used in this study was derived from the progenitor strains BN-Lx and SHR/Ola (indicated to be genetically equivalent to SHR/Lj used in this study) [25], [26]. The microsatellite marker genotypes and linkage maps for the RI panel were most recently characterized by one of our laboratories [28]. Additional microsatellite markers were identified in progenitors and mapped across the RI strains (P. Flodman et al., unpublished; O. Seda and L. Sedova, unpublished) by PCR. Marker data were correlated with SNP genotypes available through the Wellcome Trust Centre for Human Genetics STAR Rat SNP Selector (http://gscan.well.ox.ac.uk/gsBleadingEdge/rat.snp.selector.cgi).

Adult female RI rats (9–12 weeks old) of the 19 strains of the HXB/BXH set were rederived, bred, and maintained at the University of California, San Diego, and shipped to Bethesda, MD, for toxin testing and bone marrow collection. The progenitor strains and a congenic strain, SHR-Lx, were included in the analysis. Rats were acclimated for four-five days prior to experiments. For all rat LT challenge studies, female rats (130–160 g) were injected with LT (100 µg, IV) and monitored continuously for 5 h followed by a 24-h check of surviving animals. This dose of toxin represents 10× LD_100_ for the sensitive F344 and CDF rats when using the well-characterized toxin prepared in our laboratory.

### Cell culture

L929 mouse fibroblast cells were grown in Dulbecco's modified Eagle medium (DMEM) supplemented with 10% fetal bovine serum, 10 mM HEPES, and 50 µg/ml gentamicin (all obtained from Invitrogen, Carlsbad, CA) at 37°C in 5% CO_2_. Bone marrow-derived macrophages (BMDMs) were cultured in complete DMEM (as described above) with 30% L929 cell culture supernatant. BMDMs were grown for 7–9 days to allow time for differentiation before use in assays.

### Cytotoxicity assays

BMDMs were plated in 96-well plates 24 h prior to assays at 90% confluence. For basic macrophage LT sensitivity testing, cells were exposed to LT at the indicated concentrations and times. Viability was assessed by addition of MTT [3-(4,5-dimethyl-2-thiazolyl)-2,5-diphenyl tetrazolium bromide] (USB Corporation, Cleveland, OH) to a final concentration of 0.6 mg/ml in DMEM. Following 30–45 min of incubation with MTT dye, cell culture medium was removed and cells were dissolved with 0.5% SDS, 25 mM HCl in 90% isopropanol and A_570_ was measured. Percent viabilities were calculated relative to medium-treated controls.

### Statistical analyses

Statistical analyses were performed using SAS (ver. 9.1.3, SAS Institute Inc., Cary, NC). Association between genotype and phenotype across the RI strains was assessed for each marker using Fisher's exact test. Nominal p-values for the tests of association are reported without correction for multiple comparisons. The genome-wide significance of the linkage findings was assessed using MapManager QTXb20 to calculate an empiric p-value based on 10,000 permutations [41].

### *rNlrp1* sequencing

RNA was isolated from BMDMs by a TRIZOL extraction method according to manufacturer's protocol (Invitrogen). RNA was reverse transcribed using the SuperScript III First Strand Synthesis System (Invitrogen). Sequencing primers were designed to cover the full coding sequence of *rNlrp1* using the predicted *rNlrp1* mRNA sequence (GenBank accession: XM_340835) from the Rat Genome Sequencing Consortium BN rat genomic sequence data (RGSC v3.4, GenBank accession: NW_047334). The primary sequencing reactions consisted of amplifying five overlapping cDNA regions (locations indicated in Figure 3 and primer sequences are provided in Table S1). All PCR was performed with the TaKaRa Ex Taq (TAKARA Bio Inc., Otsu, Japan). PCR products were purified using PureLink PCR purification kits (Invitrogen) and sequenced on Applied Biosystems 3730xl DNA analyzers at MACROGEN USA (Rockville, MD). Additional primers and reactions were used for confirmation of specific regions and clarification of the overlaps for the primary reactions (Table S1). Sequences were assembled and analyzed using the Lasergene program suite (DNASTAR, Inc., Madison, WI). Alignments were created using ClustalX (http://www.clustal.org/) and phylogenetic trees were visualized with TreeViewX (v0.5; http://darwin.zoology.gla.ac.uk/~rpage/treeviewx/index.html). Exon structure was determined by aligning the cDNA sequences to the BN genomic data by a BLAT search (http://genome.ucsc.edu/cgi-bin/hgBlat).

### Accession codes

Amino acid sequences used in alignments and phylograms included a potential rNlrp1 paralog predicted sequence (XP_001080760.1), hNlrp1 isoform 1 (NP_127497), C57BL/6J mNlrp1a (AAZ40527), C57BL/6J mNlrp1c (AAZ40528), BALB/cJ mNlrp1b Allele 1 (AAZ40509), C57BL/6J mNlrp1b Allele 2 (AAZ40517), NOD/LtJ mNlrp1b Allele 3 (AAZ40521), DBA/2J mNlrp1b Allele 4 (AAZ40523), and CAST/EiJ mNlrp1b Allele 5 (AAZ40526). cDNA GenBank sequences for new *rNlrp1* sequences determined in this work are as follows: HM060628 (BN), HM060629 (BN-Lx), HM060630 (COP), HM060631 (Dahl/SS), HM060632 (CDF), HM060633 (LEW), HM060634 (SD), HM060635 (SHR), HM060636 (SHR/Lj), HM060637 (WIS), HM060638 (WKY), HM060639 (ZUC).

## Supporting Information

Figure S1 Full marker genotype analysis for all tested RI strains. The strain distribution patterns for the full collection of framework marker genotypes covering nearly the entire rat genome are shown [26]. Strains are indicated across the top, sorted by LT sensitivity phenotype. Genotypes are SHR-like (H; gray) or BN-Lx-like (B; white). *U* indicates unknown genotypes. Box indicates the marker most closely associated with the LT phenotype in this collection (D10Rat102).
(0.06 MB PDF)

Figure S2 *Nlrp1* paralog is not expressed in rat BMDMs. (A) RT-PCR amplification of paralog from genomic DNA using an intraexonic primer set. Primers were designed based on the predicted mRNA paralog sequence (GenBank accession: XM_001080760). (B) All lanes except the first show RT-PCR reactions using a paralog specific forward primer with a common reverse primer that spans two introns (interexonic reaction) of the predicted mRNA sequence for the paralog. Both forward and reverse primers for the interexonic paralog specific reaction were also tested and function to amplify genomic DNA when used with other paralog-specific same exon primers, ruling out any primer issues. *Nlrp1^Para^* indicates the paralog while *Nlrp1^Orig^* indicates the *rNlrp1* characterized in previous sections. (*) indicates predicted size if *Nlrp1^Para^* was transcribed. The first lane is a control RT-PCR reaction from LEW cDNA amplified with *Nlrp1^Orig^* specific forward primer and a common reverse primer producing a fragment that spans two introns (exon 3 to exon 5 of *rNlrp1*).
(0.29 MB TIF)

Figure S3 Full Nlrp1 protein alignments for all five Nlrp1 alleles. Approximate domain locations are shown above the alignments: NACHT (green), LRR (blue), and CARD (red). Red residues indicate those that differ from allele 1. Allele definitions and LT phenotypes are indicated below the alignments.
(0.02 MB PDF)

Figure S4 Phylogram of rat, mouse, and human Nlrp1 protein sequences. A neighbor-joining tree was constructed with all five rat Nlrp1 protein sequences along with the putative rNlrp1 paralog, the longest forms of hNlrp1, mNlrp1a, mNlrp1c, and all five mNlrp1b alleles. For accession codes see Materials and Methods.
(0.21 MB TIF)

Table S1 Primers used in this study.
(0.05 MB DOC)
