# Supplementary figures and images for: Susceptibility to Anthrax Lethal Toxin-Induced Rat Death Is Controlled by a Single Chromosome 10 Locus That Includes rNlrp1
Source: PLoS Pathog. 2010 May 20;6(5):e1000906. doi: 10.1371/journal.ppat.1000906 (PMC2873920; doi:10.1371/journal.ppat.1000906)

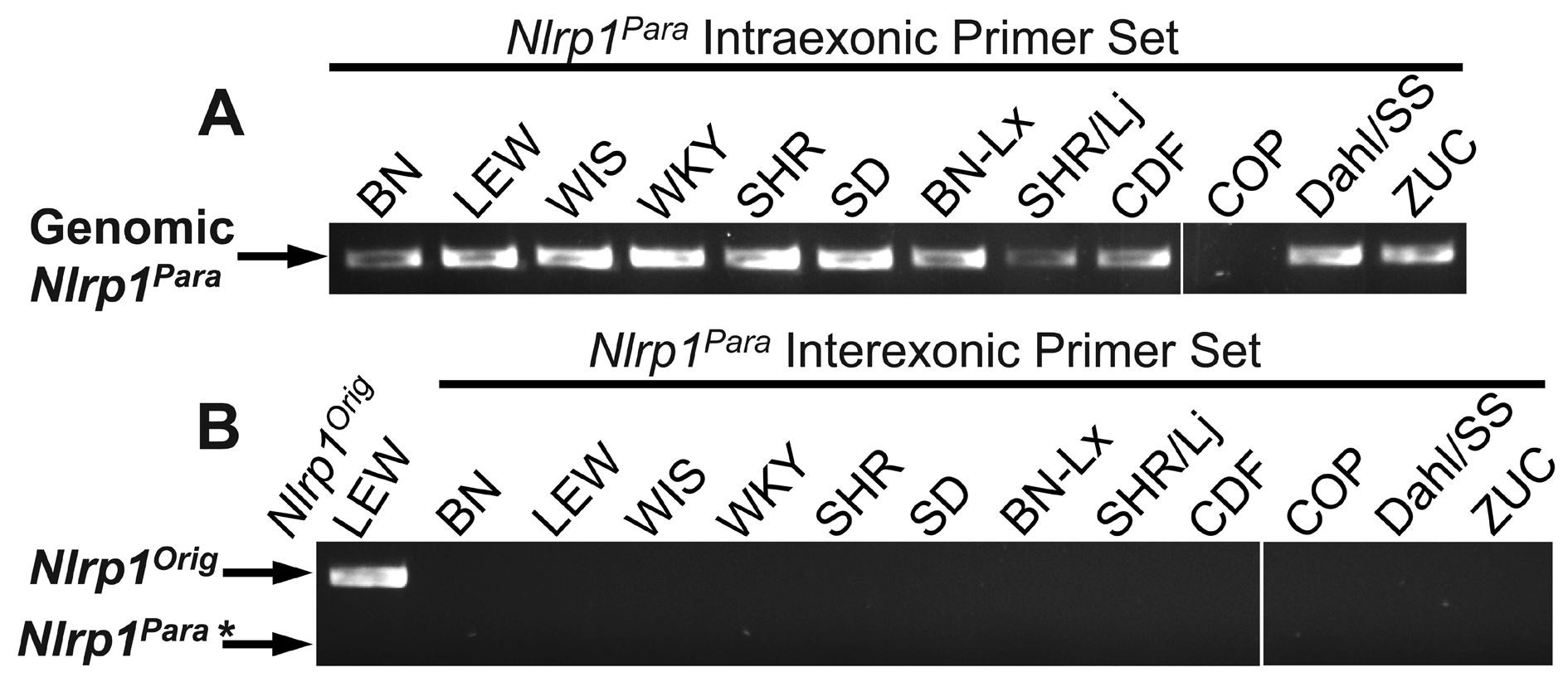

Supplement: Figure S2 — Nlrp1 paralog is not expressed in rat BMDMs. (A) RT-PCR amplification of paralog from genomic DNA using an intraexonic primer set. Primers were designed based on the predicted mRNA paralog sequence (GenBank accession: XM_001080760). (B) All lanes except the first show RT-PCR reactions using a paralog specific forward primer with a common reverse primer that spans two introns (interexonic reaction) of the predicted mRNA sequence for the paralog. Both forward and reverse primers for the interexonic paralog specific reaction were also tested and function to amplify genomic DNA when used with other paralog-specific same exon primers, ruling out any primer issues. Nlrp1Para indicates the paralog while Nlrp1Orig indicates the rNlrp1 characterized in previous sections. (*) indicates predicted size if Nlrp1Para was transcribed. The first lane is a control RT-PCR reaction from LEW cDNA amplified with Nlrp1Orig specific forward primer and a common reverse primer producing a fragment that spans two introns (exon 3 to exon 5 of rNlrp1). (0.29 MB TIF) [file ppat.1000906.s002.tif]

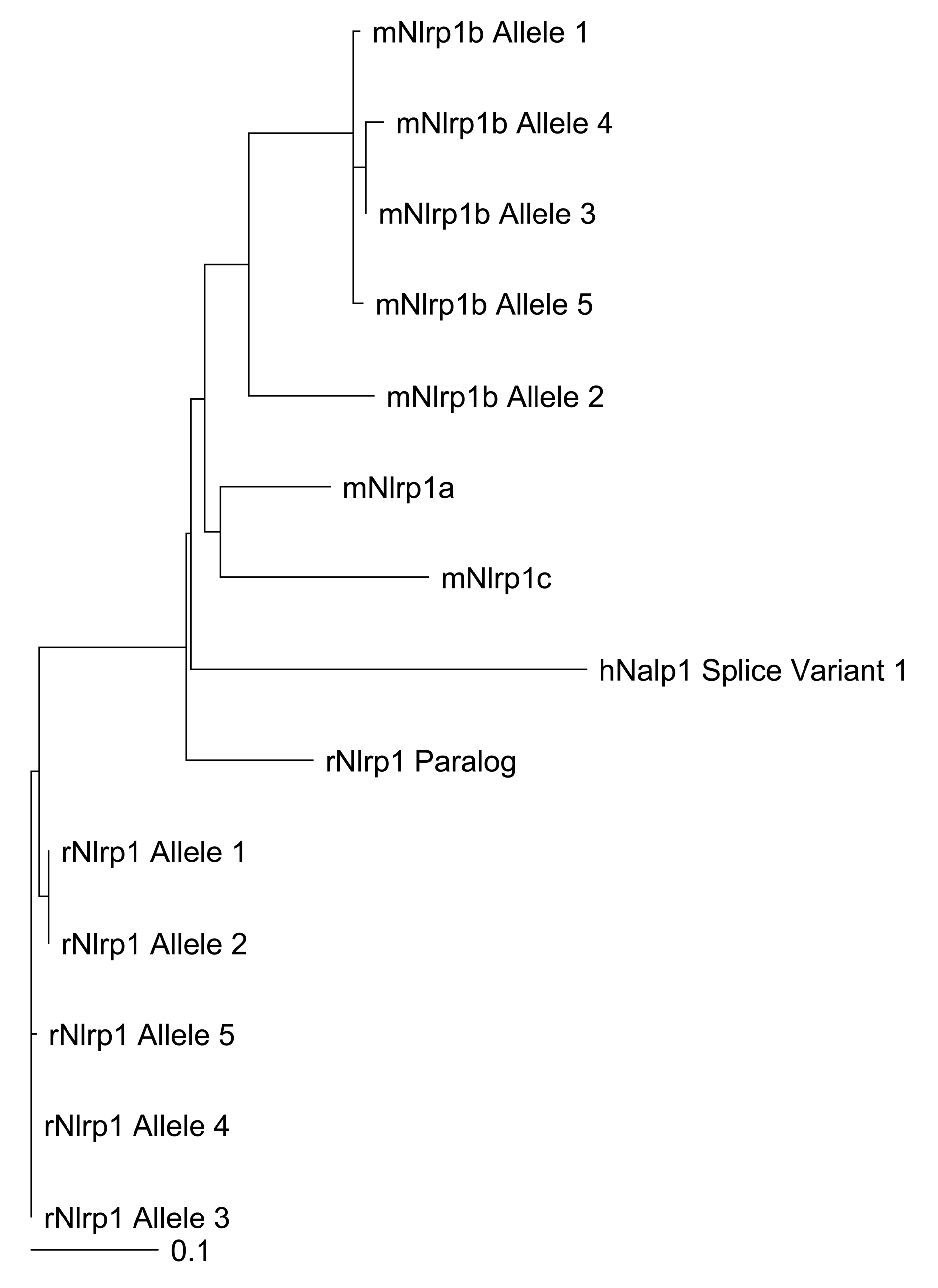

Supplement: Figure S4 — Phylogram of rat, mouse, and human Nlrp1 protein sequences. A neighbor-joining tree was constructed with all five rat Nlrp1 protein sequences along with the putative rNlrp1 paralog, the longest forms of hNlrp1, mNlrp1a, mNlrp1c, and all five mNlrp1b alleles. For accession codes see Materials and Methods. (0.21 MB TIF) [file ppat.1000906.s004.tif]
