# Supplementary material for: Susceptibility to Anthrax Lethal Toxin-Induced Rat Death Is Controlled by a Single Chromosome 10 Locus That Includes rNlrp1
Source: PLoS Pathog. 2010 May 20;6(5):e1000906. doi: 10.1371/journal.ppat.1000906 (PMC2873920; doi:10.1371/journal.ppat.1000906)
Supplement: Figure S3 — Full Nlrp1 protein alignments for all five Nlrp1 alleles. Approximate domain locations are shown above the alignments: NACHT (green), LRR (blue), and CARD (red). Red residues indicate those that differ from allele 1. Allele definitions and LT phenotypes are indicated below the alignments. (0.02 MB PDF) [file ppat.1000906.s003.pdf]

```
                                                      NACHT
Allele 1   MEESQSKQESNTRVAQHGSQQDVDPTFQTKRALEKERSKPRPRPLPRVQLQSLPGWSSTSNDVPLSQLIREMDHESRRCIHRSKKKLDRSEHISQGTIPEIYEKRKETISHTQSMEQKYL 120
Allele 2   MEESQSKQESNTRVAQHGSQQDVDPTFQTKRALEKERSKPRPRPLPRVQLQSLPGWSSTSKDVPLSQLIREMDHESRRCIHRSKKKLDRSEHISQGTIPEIYEKRKETISHTQSMEQKYL 120
Allele 3   MGESQSKQESNTRVAQHGSQQDVDPTFQTKRALERERSSPQVEQSFLGQLQSLLGWSSTSKDVPLSQLIREMDHESRRHSHQSKKKLDRSEHISEGTIPEIYEKRKETISHTQSMEQKYL 120
Allele 4   MGESQSKQESNTRVAQHGSQQDVDPTFQTKRALERERSSPQVEQSFLGQLQSLLGWSSTSKDVPLSQLIREMDHESRRHSHQSKKKLDRSEHISEGTIPEIYEKRKETISHTQSMEQKYL 120
Allele 5   MGESQSKQESNTRVAQHGSQQDVDPTFQTKRALERERSSPQVEQSFLGQLQSLLGWSSTSKDVPLSQLIREMDHESRRHSHQSKKKLDRSEHISEGTIPEIYEKRKETISHTQSMEQKYL 120

Allele 1   FQNFTKLLLLQKCCPGGSEKLVRESWHPCVPEEGGHMIEIQDLFDPNLDTEKKPQLVIIEGAAGIGKSTLARQVKRAWDEGQLYRDRFQHVFFFSCRELAQCKQLSLAELIAQGQEVPTA 240
Allele 2   FQNFTKLLLLQKCCPGGSEKLVRESWHPCVPEEGGHMIEIQDLFDPNLDTEKKPQLVIIEGAAGIGKSTLARQVKRAWDEGQLYRDRFQHVFFFSCRELAQCKQLSLAELIAQGQEVPTA 240
Allele 3   FQNFTKLLLLQKCCPGGSEKLVRESWHPCVPEEGGHMIEIQDLFDPNLDTEKKPQLVIIEGAAGIGKSTLARQVKRAWDEGQLYRDRFQHVFFFSCRELAQCKQLSLAELIAQGQEVPTA 240
Allele 4   FQNFTKLLLLQKCCPGGSEKLVRESWHPCVPEEGGHMIEIQDLFDPNLDTEKKPQLVIIEGAAGIGKSTLARQVKRAWDEGQLYRDRFQHVFFFSCRELAQCKQLSLAELIAQGQEVPTA 240
Allele 5   FQNFTKLLLLQKCCPGGSEKLVRESWHPCVPEEGGHMIEIQDLFDPNLDTEKKPQLVIIEGAAGIGKSTLARQVKRAWEEGQLYRDRFQHVFFFSCRELAQCKQLSLAELIAQGQEVLTA 240

Allele 1   PTRQILSRPEKLLFILDGIDEPAWVLEDQNPELCVHWSQAQPVHTLLGSLLGKSILPEASLMLTARTTALQKLVPSLGQPHRVEVLGFSEFERKDYFYKYFAKERNTIIDFNLIGSIPVL 360
Allele 2   PTRQILSRPEKLLFILDGIDEPAWVLEDQNPELCVHWSQAQPVHTLLGSLLGKSILPEASLMLTARTTALQKLVPSLGQPHRVEVLGFSEFERKDYFYKYFAKERNTIIDFNLIGSIPVL 360
Allele 3   PTRQILSRPEKLLFILDGIDEPAWVLEDQNPELCVHWSQAQPVHTLLGSLLGKSILPEASLMLTARTTALQKLVPSLGQPHRVEVLGFSEFERKDYFYKYFAKERNTIIDFNLIGSIPVL 360
Allele 4   PTRQILSRPEKLLFILDGIDEPAWVLEDQNPELCVHWSQAQPVHTLLGSLLGKSILPEASLMLTARTTALQKLVPSLGQPHRVEVLGFSEFERKDYFYKYFAKERNTIIDFNLIGSIPVL 360
Allele 5   PTRQILSRPEKLLFILDGIDEPAWVLEDQNPELCVHWSQAQPVHTLLGSLLGKSILPEASLMLTARTTALQKLIPSLGQPHRVEVLGFSEFERKDYFYKYFAKERNTIIDFNLIGSIPVL 360

Allele 1   LTLCEVPWVCWLLCTCLEKQMQQGEVLSLTSQTTTALCLKYLSLTIPGQHLSTQLRTLCSLAAEGICQRRTLFSKSDLCKQGLAEDAIATFLKIGVLQRQPSSLSYSFAHLCLQEFFAAM 480
Allele 2   LTLCEVPWVCWLLCTCLEKQMQQGEVLSLTSQTTTALCLKYLSLTIPGQHLSTQLRTLCSLAAEGICQRRTLFSKSDLCKQGLAEDAIATFLKIGVLQRQPSSLSYSFAHLCLQEFFAAM 480
Allele 3   LTLCEVPWVCWLLCTCLEKQMQQGEVLSLTSQTTTALCLKYLSLTIPGQHLSTQLRTLCSLAAEGICQRRTLFSKSDLCKQGLAEDAIATFLKIGVLQRQPSSLSYSFAHLCLQEFFAAM 480
Allele 4   LTLCEVPWVCWLLCTCLEKQMQQGEVLSLTSQTTTALCLKYLSLTIPGQHLSTQLRTLCSLAAEGICQRRTLFSKSDLCKQGLAEDAIATFLKIGVLQRQPSSLSYSFAHLCLQEFFAAM 480
Allele 5   LTLCEVPWVCWLLCTCLEKQMQQGEVLSLTSQTTTALCLKYLSLTIPGQHLSTQLRTLCSLAAEGICQRRTLFSKSDLCKQGLAEDAIATFLKIGVLQRQPSSLSYSFAHLCLQEFFAAM 480

Allele 1   SYILEDSEEAHGDMGNDRTVETLVERYGRQNLFEAPTVRFLLGLLNTREMREMENIFACKFPWETKLKLLQSIIGEPFCQPCHLGLFHCLYENQEEELLTETMLCFPLTASGPNHMEATV 600
Allele 2   SYILEDSEEAHGDMGNDRTVETLVERYGRQNLFEAPTVRFLLGLLNTREMREMENIFACKFPWETKLKLLQSIIGEPFCQPCHLGLFHCLYENQEEELLTETMLCFPLTASGPNHMEATV 600
Allele 3   SYILEDSEEAHGDMGNDRTVETLVERYGRQNLFEAPTVRFLLGLLNTREMREMENIFACKFPWETKLKLLQSIIGEPFCQPCHLGLFHCLYENQEEELLTETMLCFPLTASGPNHMEATV 600
Allele 4   SYILEDSEEAHGDMGNDRTVETLVERYGRQNLFEAPTVRFLLGLLNTREMREMENIFACKFPWETKLKLLRSIIGEPFCQPCHLGLFHCLYENQEEELLTETMLCFPLTASGPNHMEATV 600
Allele 5   SYILEDSEEARGDMGNDRTVETLVERYGRQNLFEAPTVRFLLGLLNTREMREMENIFACKFPWKTKLKLLRSIVGEPFCQPCHLGLFHCLYENQEEELLTETMLCFPLTASGPNHMEATV 600

                                                                LRR
Allele 1   FQTNVKRLVIQTDMELMVVTFCITFSHVRSLRLKGKGQQEYKLTAPAMVLYRWTPISEASWKVLFSNLKCTRNLEELDLSGNPLSYSAVRSLCTALRQPGCRLKTLWLVDCGLTSRCCSF 720
Allele 2   FQTNVKRLVIQTDMELMVVTFCITFSHVRSLRLKGKGQQEYKLTAPAMVLYRWTPISEASWKVLFSNLKCTRNLEELDLSGNPLSYSAVRSLCTALRQPGCRLKTLWLVDCGLTSRCCSF 720
Allele 3   FQTNVKRLVIQTDMELMVVTFCITFSHVRSLRLKGKGQQEYKLTAPAMVLYRWTPISEASWKVLFSNLKCTRNLEELDLSGNPLSYSAVRSLCTALRQPGCRLKTLWLVDCGLTSRCCSF 720
Allele 4   FQTNVKRLVIQTDMELMVVTFCITFSHVRSLRLKGKGQQEYKLTAPAMVLYRWTPISEASWKVLFSNLKCTRNLEELDLSGNPLSYSAVRSLCTALRQPGCRLKTLWLVDCGLTSRCCSF 720
Allele 5   FQTNVKRLVIQTDMELMVVTFCITFSHVRSLRLKGKGQQEYKLTAPAMVLYRWTPISEASWKVLFSNLKCTRNLEELDLSGNPLSYSAVRSLCTALRQPGCRLKTLWLVDCGLTSRCCSF 720

Allele 1   LASMLSAHSRLAELDLRLNDLGDNGVRQLCEGLRNPACNLSILRLDQASLSEQVITELRALETKNPKLFISSTWMSHMTMPTENTDGEESLTSSKQQQQQSGDKHMEPLGTDDDFWGPSG 840
Allele 2   LASMLSAHSRLAELDLRLNDLGDNGVRQLCEGLRNPACNLSILRLDQASLSEQVITELRALETKNPKLFISSTWMSHMTMPTENTDGEESLTSSKQQQQQSGDKHMEPLGTDDDFWGPSG 840
Allele 3   LASMLSAHSRLAELDLRLNDLGDNGVRQLCEGLRNPACNLSILRLDQASLSEQVITELRALETKNPKLFISSTWMSHMTMPTENTDGEESLTSSKQQQQQSGDKHMEPLGTDDDFWGPSG 840
Allele 4   LASMLSAHSRLAELDLRLNDLGDNGVRQLCEGLRNPACNLSILRLDQASLSEQVITELRALETKNPKLFISSTWMSHMTMPTENTDGEESLTSSKQQQQQSGDKHMEPLGTDDDFWGPSG 840
Allele 5   LASMLSAHSRLAELDLRLNDLGDNGVRQLCEGLRNPACNLSILRLDQASLSEQVITELRALETKNPKLFISSTWMSHMTMPTENTDGEESLTSSKQQQQQSGDKHMEPLGTDDDFWGPSG 840

Allele 1   PVSTEVVDRERNLYRVRLPMAGSYHCPSTGLHFVVTRAVTIEIGFCAWSQFLHETPLQHSHMVAGPLFDIKAEHGAVTAVCLPHFVSLQEGKVDSSLFHVAHFQDHGMVLETPARVEPHF 960
Allele 2   PVSTEVVDRERNLYRVRLPMAGSYHCPSTGLHFVVTRAVTIEIGFCAWSQFLHETPLQHSHMVAGPLFDIKAEHGAVTAVCLPHFVSLQEGKVDSSLFHVAHFQDHGMVLETPARVEPHF 960
Allele 3   PVSTEVVDRERNLYRVRLPMAGSYHCPSTGLHFVVTRAVTIEIGFCAWSQFLHETPLQHSHMVAGPLFDIKAEHGAVTAVCLPHFVSLQEGKVDSSLFHVAHFQDHGMVLETPARVEPHF 960
Allele 4   PVSTEVVDRERNLYRVRLPMAGSYHCPSTGLHFVVTRAVTIEIGFCAWSQFLHETPLQHSHMVAGPLFDIKAEHGAVTAVCLPHFVSLQEGKVDSSLFHVAHFQDHGMVLETPARVEPHF 960
Allele 5   PVSTEVVDRERNLYRVRLPMAGSYHCPSTGLHFVVTRAVTIEIGFCAWSQFLHETPLQHSHMVAGPLFDIKAEHGAVTAVCLPHFVSLQEGKVDSSLFHVAHFQDHGMVLETPARVEPHF 960

Allele 1   AVLENPSFSPMGVLLRMIPAVGHFIPITSITLIYYRLYLEDITFHLYLVPNDCTIRKAIDEEELKFQFVRINKPPPVDALYVGSRYIVSSSKEVEILPKELELCYRSPRESQLFSEIYVG 1080
Allele 2   AVLENPSFSPMGVLLRMIPAVGHFIPITSITLIYYRLYLEDITFHLYLVPNDCTIRKAIDEEELKFQFVRINKPPPVDALYVGSRYIVSSSKEVEILPKELELCYRSPRESQLFSEIYVG 1080
Allele 3   AVLENPSFSPMGVLLRMIPAVGHFIPITSITLIYYRLYLEDITFHLYLVPNDCTIRKAIDEEELKFQFVRINKPPPVDALYVGSRYIVSSSKEVEILPKELELCYRSPRESQLFSEIYVG 1080
Allele 4   AVLENPSFSPMGVLLRMIPAVGHFIPITSITLIYYRLYLEDITFHLYLVPNDCTIRKAIDEEELKFQFVRINKPPPVDALYVGSRYIVSSSKEVEILPKELELCYRSPRESQLFSEIYVG 1080
Allele 5   AVLENPSFSPMGVLLRMIPAVGHFIPITSITLIYYRLYLEDITFHLYLVPNDCTIRKAIDEEELKFQFVRINKPPPVDALYVGSRYIVSSSKEVEILPKELELCYRSPRESQLFSEIYVG 1080

                                             CARD
Allele 1   NIGSGINLQLTDKKYMNLIWEALLKPGDLRPALPRMASAPKDAPALLHFVDQHREQLVARVTSVDPLLDKLHGLVLSEEDYETVRAEATNQDKMRKLFRGSRSWSWDCKDHFYQALKETH 1200
Allele 2   NIGSGINLQLTDKKYMNLIWEALLKPGDLRPALPRMASAPKDAPALLHFVDQHREQLVARVTSVDPLLDKLHGLVLSEEDYETVRAEATNQDKMRKLFRGSRSWSWDCKDHFYQALKETH 1200
Allele 3   NIGSGINLQLTDKKYMNLIWEALLKPGDLRPALPRMASAPKDAPALLHFVDQHREQLVARVTSVDPLLDKLHGLVLSEEDYETVRAEATNQDKMRKLFRGSRSWSWDCKDHFYQALKETH 1200
Allele 4   NIGSGINLQLTDKKYMNLIWEALLKPGDLRPALPRMASAPKDAPALLHFVDQHREQLVARVTSVDPLLDKLHGLVLSEEDYETVRAEATNQDKMRKLFRGSRSWSWDCKDHFYQALKETH 1200
Allele 5   NIGSGINLQLTDKKYMNLIWEALLKPGDLRPALPRMASAPKDAPALLHFVDQHREQLVARVTSVDPLLDKLHGLVLSEEDYETVRAEATNQDKMRKLFRGSRSWSWDCKDHFYQALKETH 1200

Allele 1   PHLIMDLLEKSGGVSVRL 1218
Allele 2   PHLIMDLLEKSGGVSVRL 1218
Allele 3   PHLIMDLLEKSGGVSVRL 1218
Allele 4   PHLIMDLLEKSGGVSVRL 1218
Allele 5   PHLIMDLLEKSGGVSVRL 1218
```

Allele 1: LT-Sensitive (Brown Norway, Wistar, Sprague Dawley, BN-Lx, Dahl/SS)

Allele 2: LT-Sensitive (Fischer CDF)

Allele 3: LT-Resistant (Zucker-Lean)

Allele 4: LT-Resistant (Copenhagen)

Allele 5 LT-Resistant (Lewis, Wistar-Kyoto, SHR, SHR/Lj)
