# Supplementary material for: Susceptibility to Anthrax Lethal Toxin-Induced Rat Death Is Controlled by a Single Chromosome 10 Locus That Includes rNlrp1
Source: PLoS Pathog. 2010 May 20;6(5):e1000906. doi: 10.1371/journal.ppat.1000906 (PMC2873920; doi:10.1371/journal.ppat.1000906)
Supplement: Table S1 — Primers used in this study. (0.05 MB DOC) [file ppat.1000906.s005.doc]

| **Table S1. Primers used in this study** | |
| --- | --- |
| **Primary Sequencing Reactions (see Figure 3)** | |
| *rNlrp1* Sequencing Piece 1 | F: GAGAGTCTTGATGCACAAACTTCTCAGAG  R: CAGGCTCTCTTCACCTGCCTGGCCAG |
| *rNlrp1* Sequencing Piece 2 | F: CATATGATTGAGATCCAAGACTTAT  R: GCTTCCTCACTATCCTCCAAGATGT |
| *rNlrp1* Sequencing Piece 3 | F: TCTGAGCTACAGCTTTGCCCACTTGT  R: ATCCATGTGCTGGAGATGAACAGCT |
| *rNlrp1* Sequencing Piece 4 | F: CAGGCCTCTCTCAGTGAGCAGGTGA  R: CACGATATAGCGGGAACCAACATAAAG |
| *rNlrp1* Sequencing Piece 5 | F: GGACATCACCTTTCACCTTTACCTG  R: CTGGGTTACACATGGAGAAACTAAG |
| **Secondary Sequencing Reactions*** | |
| *rNlrp1* Sequencing Piece 1b (at 5’ end of CDS) | F: ATGGAAGAATCTCAGTCCAAGCAG  R: CAGGCTCTCTTCACCTGCCTGGCCAG |
| *rNlrp1* Sequencing Piece 5b (at 3’ end of CDS) | F: GGACATCACCTTTCACCTTTACCTG  R: CAGGATGTCAGAGTCTAACAGAGA |
| Sequence confirmation 5’ end (only used in the sequencer) | R: ATAAGTCTTGGATCTCAATCATATG |
| Sequence clarification piece 2 to piece 3 gap (only used in the sequencer) | F: CTGTTCAGTAAAAGTGACCTCTGTA |
| Sequence clarification piece 3 to piece 2 gap (only used in the sequencer) | R: GACAGTGGAACAAACCCAGATGACAT |
| Sequence clarification piece 3 to piece 4 gap (only used in the sequencer) | F: ATGTCATCTGGGTTTGTTCCACTGTC |
| Sequence clarification piece 4 to piece 3 gap (only used in the sequencer) | R: ATGGGCAATCGCACTCGGTACAGGT |
| **Paralog Searching Reactions (see Figure S2)** | |
| *rNlrp1^Para^*† Intraexonic Primer Set (within a predicted exon that is highly homologous to *rNlrp1* Exon 3) | F: CATATGATTGAGATCCAAGACTTAT  R: CAGACACGATGTAGCGGGAGCCGATGTACAGGCT |
| *rNlrp1^Orig^*† Interexonic Primer Set (Spans exon 3 to exon 5 according to Figure 3) (Reverse primer is common between *rNlrp1^Orig^* and *rNlrp1^Para^*) | F: TCTGAGCTACAGCTTTGCCCACTTGT  R: CAGCATCTGGATGTGAGGCCACA |
| *rNlrp1^Para^*† Specific Interexonic Primer Set (predicted to span 2 excised introns if cDNA for paralog exists) (Reverse primer is common between *rNlrp1^Orig^* and *rNlrp1^Para^*, Forward primer is paralog specific‡) | F: ATGATCTTCAAGGAATAATAGTGCCTGACA  R: CAGCATCTGGATGTGAGGCCACA |
| * For simplicity the locations of these primers were not noted in Figure 3.  † *rNlrp1^Orig^* and *rNlrp1^Para^* refer to the sequenced full length *rNlrp1* reported in this paper and its putative tandem paralog, respectively.  ‡ Note that this primer has been tested with another paralog specific primer within an intronic region and works, suggesting that both primers do work well separately with the paralog contaminating DNA in the cDNA prep. | |
