# Supplementary material for: Susceptibility to Anthrax Lethal Toxin-Induced Rat Death Is Controlled by a Single Chromosome 10 Locus That Includes rNlrp1
Source: PLoS Pathog. 2010 May 20;6(5):e1000906. doi: 10.1371/journal.ppat.1000906 (PMC2873920; doi:10.1371/journal.ppat.1000906)
Supplement: Figure S1 — Full marker genotype analysis for all tested RI strains. The strain distribution patterns for the full collection of framework marker genotypes covering nearly the entire rat genome are shown [26]. Strains are indicated across the top, sorted by LT sensitivity phenotype. Genotypes are SHR-like (H; gray) or BN-Lx-like (B; white). U indicates unknown genotypes. Box indicates the marker most closely associated with the LT phenotype in this collection (D10Rat102). (0.06 MB PDF) [file ppat.1000906.s001.pdf]

| MARKERS | LT-Resistant | | | | | | | | | LT-Sensitive | | | | | | | | | |
|---|---|---|---|---|---|---|---|---|---|---|---|---|---|---|---|---|---|---|---|
| **Chr. 1** | **1H** | **4H** | **7H** | **25H** | **27H** | **6B** | **O8B** | **11B** | **13B** | **2H** | **3H** | **10H** | **15H** | **17H** | **18H** | **23H** | **29H** | **31H** | **10B** |
| D1Rat327 | B | H | B | H | B | H | H | H | B | H | B | H | B | B | B | B | B | B | B |
| D1Rat186 | H | H | B | H | H | H | H | H | B | H | B | H | B | B | B | H | B | B | B |
| D1Mgh2 | H | H | B | H | B | H | B | B | B | H | B | H | H | H | B | B | B | B | B |
| D1Rat15 | H | H | B | H | B | H | B | B | B | B | B | H | H | H | B | B | B | B | B |
| D1Rat18 | H | H | H | H | H | H | B | B | B | B | B | H | B | H | H | B | B | H | B |
| D1Rat256 | H | H | H | H | H | H | H | B | B | H | H | H | H | H | H | B | B | H | B |
| D1Pas1 | B | H | H | H | B | H | *U* | B | B | H | H | B | H | B | H | B | B | H | B |
| D1Rat24 | H | H | H | H | B | H | H | H | B | H | H | H | H | B | H | B | B | B | H |
| D1Rat212 | H | H | H | H | B | H | B | H | B | H | H | H | H | B | H | H | B | B | H |
| D1Rat266 | B | H | H | H | B | H | H | H | B | H | H | B | H | H | H | H | B | H | B |
| D1Rat30 | B | H | H | H | H | H | B | H | B | H | H | B | H | H | H | H | B | H | B |
| D1Rat268 | B | H | H | H | B | H | B | H | B | H | B | H | H | H | H | H | H | H | B |
| D1Rat35 | B | H | H | B | B | B | H | H | B | H | H | H | H | H | H | B | H | H | B |
| D1Rat270 | B | H | H | B | B | B | B | H | B | H | H | H | H | B | B | B | H | H | B |
| D1Rat42 | H | H | B | B | B | B | B | H | B | H | B | H | B | B | B | B | B | B | H |
| D1Rat287 | H | H | B | B | B | B | B | H | B | H | B | B | B | H | B | H | H | B | B |
| D1Rat292 | H | H | B | H | B | B | H | H | B | H | H | B | B | H | H | B | H | H | B |
| D1Rat293 | H | H | B | H | B | H | H | H | B | H | B | B | B | H | H | B | H | B | B |
| D1Rat71 | B | H | B | H | B | H | H | H | B | B | B | B | B | H | H | H | H | B | B |
| D1Rat296 | H | H | H | B | B | B | H | H | B | H | B | B | B | H | H | H | H | B | B |
| D1Mit34 | B | H | H | B | B | B | H | H | H | H | H | B | B | H | B | B | H | B | B |
| D1Rat77 | B | H | H | B | B | B | B | H | H | H | B | B | B | B | B | B | H | B | B |
| D1Rat304 | B | H | H | H | B | B | B | H | B | H | H | B | B | B | B | B | H | H | B |
| D1Rat81 | H | H | H | H | B | B | B | H | B | H | H | B | B | H | H | B | H | H | B |
| D1Rat225 | H | H | H | H | B | B | B | H | B | H | H | B | H | H | H | B | H | H | B |
| D1Mit14 | H | H | H | H | B | B | *U* | *U* | B | H | H | H | H | H | H | B | H | H | B |

| **Chr. 2** | **1H** | **4H** | **7H** | **25H** | **27H** | **6B** | **O8B** | **11B** | **13B** | **2H** | **3H** | **10H** | **15H** | **17H** | **18H** | **23H** | **29H** | **31H** | **10B** |
|---|---|---|---|---|---|---|---|---|---|---|---|---|---|---|---|---|---|---|---|
| D2Rat189 | B | B | H | H | B | B | B | H | H | B | H | B | H | B | H | B | B | H | H |
| D2Rat124 | B | B | B | B | B | B | B | H | H | B | B | B | H | B | H | B | B | H | H |
| D2Rat94 | H | H | B | B | B | B | B | H | H | H | B | H | H | B | H | B | B | H | H |
| D2Rat11 | H | H | H | B | B | B | B | H | H | B | H | H | H | B | B | B | B | H | H |
| D2Rat201 | B | B | H | B | B | B | B | H | H | H | B | H | H | H | B | H | B | H | H |
| D2Rat320 | B | B | B | B | B | B | H | H | H | H | H | H | H | H | B | H | B | H | H |
| D2Mit6 | B | B | B | B | B | B | *U* | H | H | B | B | H | H | H | B | H | B | H | B |
| D2Rat95 | B | B | H | B | B | H | H | H | H | H | B | H | H | H | B | H | B | H | B |
| D2Rat24 | B | B | H | B | B | H | H | H | H | H | B | H | H | H | B | B | B | H | B |
| D2Rat147 | B | H | H | B | B | H | B | H | H | H | B | H | H | H | B | B | B | H | H |
| D2Rat221 | H | H | H | B | B | H | B | H | H | H | B | H | H | H | B | B | B | H | H |
| D2Rat152 | H | H | B | B | B | H | B | B | H | B | H | H | H | H | B | B | B | B | H |
| D2Rat228 | B | H | B | H | B | B | B | B | H | B | H | H | B | H | B | B | H | B | H |
| D2Rat236 | B | H | B | H | B | B | B | H | H | B | B | H | H | H | B | B | H | B | H |
| D2Rat62 | B | B | B | H | B | B | B | H | H | H | H | H | B | H | B | H | B | B | H |
| D2Rat247 | B | B | B | H | B | B | B | H | B | H | B | H | B | B | B | H | H | B | H |
| D2Rat66 | H | H | B | H | B | B | B | H | B | B | B | H | B | B | B | H | H | B | H |
| D2Rat67 | H | H | B | H | B | B | B | B | B | H | B | H | B | B | B | H | H | B | H |
| D2Rat69 | H | H | B | H | B | B | H | B | B | H | B | H | B | B | H | H | H | H | H |
| D2Rat70 | H | H | B | B | B | B | B | B | B | B | B | H | B | B | H | B | H | H | H |

| **Chr. 3** | **1H** | **4H** | **7H** | **25H** | **27H** | **6B** | **O8B** | **11B** | **13B** | **2H** | **3H** | **10H** | **15H** | **17H** | **18H** | **23H** | **29H** | **31H** | **10B** |
|---|---|---|---|---|---|---|---|---|---|---|---|---|---|---|---|---|---|---|---|
| D3Rat194 | H | H | H | B | B | H | H | B | B | B | H | H | H | H | H | B | H | H | H |
| D3Rat53 | B | B | H | B | B | H | H | B | B | B | B | H | B | H | H | B | B | H | H |
| D3Rat82 | H | B | B | B | B | B | H | B | B | B | B | H | B | H | H | B | B | H | H |
| D3Rat188 | H | H | B | B | B | B | B | B | B | H | H | H | H | H | H | B | B | H | B |
| D3Rat185 | H | H | B | B | B | B | H | B | B | H | H | H | H | H | H | B | H | H | B |
| D3Rat183 | B | B | H | B | B | B | H | B | B | H | H | H | H | H | H | B | H | H | H |
| D3Rat180 | H | B | B | B | B | H | B | H | B | H | H | H | H | B | H | B | H | H | H |
| D3Rat35 | H | B | B | B | B | H | B | H | B | H | H | H | H | B | B | B | H | B | B |
| D3Rat173 | H | B | B | B | B | H | B | B | B | H | H | B | H | B | B | B | H | B | B |
| D3Rat166 | H | B | B | B | B | B | B | B | B | H | H | B | B | B | H | B | H | H | B |
| D3Rat20 | H | B | B | H | B | B | B | B | B | H | B | B | B | B | H | B | H | H | H |
| D3Rat159 | H | B | B | H | B | H | B | B | B | H | B | B | B | H | H | B | H | B | H |
| D3Rat157 | B | B | B | H | B | H | H | B | B | H | H | B | H | H | H | B | H | B | H |
| D3Rat6 | B | B | B | H | B | H | H | B | H | H | H | H | H | H | H | H | H | H | H |
| D3Rat143 | B | B | B | H | B | H | H | B | B | H | H | B | B | H | H | B | H | H | H |
| D3Rat132 | B | B | H | H | B | B | H | H | H | H | H | B | B | B | H | B | H | H | H |
| D3Rat1 | B | B | H | H | B | B | H | H | H | B | H | B | B | B | H | B | H | H | H |

| **Chr. 4** | **1H** | **4H** | **7H** | **25H** | **27H** | **6B** | **O8B** | **11B** | **13B** | **2H** | **3H** | **10H** | **15H** | **17H** | **18H** | **23H** | **29H** | **31H** | **10B** |
|---|---|---|---|---|---|---|---|---|---|---|---|---|---|---|---|---|---|---|---|
| D4Rat4 | H | H | B | H | H | H | B | H | H | H | B | H | B | H | H | H | B | H | B |
| D4Rat7 | H | H | B | H | B | H | B | H | H | H | B | H | B | H | H | H | H | H | B |
| D4Rat10 | H | H | B | H | B | H | B | H | H | B | B | H | B | H | H | B | H | H | B |
| D4Rat151 | B | H | B | H | H | H | B | H | H | B | B | H | B | H | H | B | H | H | B |
| D4Rat153 | B | H | H | H | B | H | B | H | H | H | B | H | B | H | H | H | H | B | B |
| D4Rat102 | B | H | H | B | B | H | B | H | H | H | B | B | B | H | H | B | H | B | B |
| D4Rat168 | B | H | B | H | B | H | B | H | H | H | B | B | B | H | H | B | H | B | B |
| D4Rat169 | B | H | B | H | B | H | B | H | H | H | B | B | B | H | H | H | H | B | B |
| D4Rat35 | B | B | B | H | H | B | B | H | H | H | B | B | B | H | H | H | H | B | B |
| D4Rat176 | B | B | B | H | H | H | B | H | H | H | B | B | B | H | H | B | B | B | B |
| D4Rat44 | B | B | B | H | H | H | H | H | H | H | B | B | B | H | H | H | B | B | B |
| D4Rat58 | H | H | B | H | H | H | H | B | H | H | B | B | B | H | H | H | B | H | B |
| D4Rat240 | B | H | H | H | H | H | H | H | H | H | B | B | B | H | *U* | H | H | H | B |
| D4Rat198 | H | H | H | H | H | H | H | B | H | H | B | B | B | H | H | H | H | H | B |
| D4Rat202 | H | H | B | H | H | H | B | H | B | H | B | B | B | H | H | H | H | H | B |
| D4Rat68 | H | H | H | H | H | H | B | B | B | H | B | B | B | H | B | H | H | H | B |

| **Chr. 5** | **1H** | **4H** | **7H** | **25H** | **27H** | **6B** | **O8B** | **11B** | **13B** | **2H** | **3H** | **10H** | **15H** | **17H** | **18H** | **23H** | **29H** | **31H** | **10B** |
|---|---|---|---|---|---|---|---|---|---|---|---|---|---|---|---|---|---|---|---|
| D5Mgh17 | H | B | H | B | B | B | H | B | B | B | H | B | H | B | B | B | H | H | B |
| D5Rat188 | H | B | B | B | B | B | H | H | B | B | H | B | H | H | H | B | H | H | H |
| D5Rat126 | H | B | H | B | B | B | H | H | B | B | H | B | H | H | H | H | H | H | H |
| D5Rat6 | H | H | H | H | B | B | H | H | B | B | H | B | H | H | H | H | H | H | H |
| D5Rat135 | B | H | H | H | B | B | H | H | B | B | H | B | H | B | H | H | H | H | H |
| D5Rat228 | B | B | H | H | B | H | H | H | B | B | H | B | H | B | H | H | H | H | B |
| D5Rat144 | B | B | H | H | B | H | H | H | B | B | H | B | H | H | H | H | B | H | B |
| D5Rat147 | B | H | H | H | B | H | H | H | B | H | H | B | H | H | H | H | B | H | B |
| D5Mit5 | H | H | H | H | B | H | *U* | H | B | H | H | H | H | H | H | H | B | H | B |
| D5Rat158 | H | B | H | H | B | B | H | H | B | H | H | H | H | B | H | H | B | H | H |
| D5Rat169 | H | B | B | H | H | B | H | B | B | H | H | H | H | B | H | H | B | H | H |
| D5Rat34 | H | B | B | B | H | B | H | B | H | H | H | H | H | H | H | H | H | H | H |
| D5Rat63 | H | B | B | H | H | H | B | B | H | H | H | H | H | B | H | H | H | H | B |
| D5Rat93 | B | B | B | H | H | H | B | H | H | B | H | B | H | B | B | B | H | H | B |
| D5Rat45 | B | H | B | H | H | H | B | H | H | B | H | B | H | B | B | B | H | H | B |
| D5Rat245 | B | H | B | H | H | H | B | H | H | B | H | H | H | H | B | B | B | B | B |

| **Chr. 6** | **1H** | **4H** | **7H** | **25H** | **27H** | **6B** | **O8B** | **11B** | **13B** | **2H** | **3H** | **10H** | **15H** | **17H** | **18H** | **23H** | **29H** | **31H** | **10B** |
|---|---|---|---|---|---|---|---|---|---|---|---|---|---|---|---|---|---|---|---|
| D6Rat46 | B | B | H | H | B | B | B | B | B | H | B | H | B | H | B | B | B | H | H |
| D6Rat80 | B | B | H | H | B | B | B | B | B | B | B | H | B | B | B | B | B | H | H |
| D6Rat147 | H | H | H | H | B | B | B | B | B | B | B | H | B | B | B | B | H | H | H |
| D6Rat171 | H | H | H | H | B | B | B | B | B | B | B | H | B | B | B | B | H | H | B |
| D6Rat84 | H | H | H | B | B | B | B | H | B | B | B | H | B | B | B | H | H | H | B |
| D6Rat28 | H | H | H | B | B | B | B | H | B | B | B | B | H | B | B | B | H | H | B |
| D6Rat132 | B | H | H | B | B | B | H | H | B | B | B | B | H | B | B | H | B | H | H |
| D6Rat165 | B | H | B | B | H | H | H | H | B | H | B | B | B | B | B | H | B | H | H |
| D6Rat87 | B | H | B | H | H | H | H | H | B | B | B | H | B | B | B | H | B | H | H |
| D6Rat88 | H | H | B | H | H | H | H | H | B | H | B | B | H | B | B | H | B | H | H |
| D6Rat117 | H | H | B | B | H | H | H | B | B | H | B | B | B | B | B | H | H | H | H |
| D6Rat11 | H | H | H | H | H | H | H | B | H | H | B | B | B | B | B | H | H | B | H |
| D6Rat184 | H | H | B | H | H | H | H | B | H | H | B | B | B | B | B | H | B | B | H |
| D6Rat111 | H | B | B | H | H | H | H | B | H | H | B | H | B | B | B | H | B | B | H |
| D6Rat101 | *U* | B | B | H | H | H | H | H | H | H | B | B | B | B | B | H | B | B | B |
| D6Rat1 | H | B | B | B | H | B | H | H | H | H | B | B | B | H | B | H | B | B | B |
| D6Rat3 | H | B | B | B | H | H | H | H | H | H | B | B | B | H | B | H | B | B | B |

| **Chr. 7** | **1H** | **4H** | **7H** | **25H** | **27H** | **6B** | **O8B** | **11B** | **13B** | **2H** | **3H** | **10H** | **15H** | **17H** | **18H** | **23H** | **29H** | **31H** | **10B** |
|---|---|---|---|---|---|---|---|---|---|---|---|---|---|---|---|---|---|---|---|
| D7Rat35 | B | H | B | B | H | H | B | H | B | B | H | B | H | B | H | B | H | H | B |
| D7Rat32 | B | H | B | B | H | H | B | H | B | B | H | B | H | B | B | B | H | H | B |
| D7Rat152 | B | H | B | B | B | H | H | H | B | B | H | H | H | B | B | B | B | H | B |
| D7Mit17 | B | H | B | B | H | H | *U* | H | B | B | H | B | H | B | H | B | B | H | B |
| D7Rat181 | H | H | H | B | B | H | B | H | B | *U* | B | H | B | B | H | B | B | H | B |
| D7Rat103 | H | H | H | B | B | H | B | H | B | B | H | H | H | B | H | H | B | H | B |
| D7Rat25 | H | H | H | B | B | B | B | H | B | B | B | B | H | B | H | H | B | B | B |
| D7Rat110 | H | B | H | B | B | B | H | H | B | B | H | B | H | B | H | H | B | B | B |
| D7Mit5 | B | B | H | B | H | B | B | H | B | B | H | B | H | B | H | H | B | B | B |
| D7Rat112 | B | H | B | B | H | B | B | H | B | H | H | B | H | B | B | H | H | B | B |
| D7Rat19 | B | H | B | B | B | B | B | H | B | H | H | B | H | B | H | H | H | H | H |
| D7Rat133 | B | H | H | H | H | B | B | H | B | H | B | B | H | H | H | H | H | H | H |
| D7Rat102 | H | H | H | H | B | B | B | B | B | *U* | H | B | H | B | B | H | H | H | H |
| D7Rat196 | *U* | H | H | H | B | H | B | H | H | B | H | B | H | B | B | H | H | H | H |
| D7Rat4 | B | H | B | H | B | H | H | H | H | B | H | B | H | H | H | B | H | H | H |
| D7Rat2 | B | H | B | H | B | H | H | H | H | H | H | B | H | H | H | B | H | H | H |

| **Chr. 8** | **1H** | **4H** | **7H** | **25H** | **27H** | **6B** | **O8B** | **11B** | **13B** | **2H** | **3H** | **10H** | **15H** | **17H** | **18H** | **23H** | **29H** | **31H** | **10B** |
|---|---|---|---|---|---|---|---|---|---|---|---|---|---|---|---|---|---|---|---|
| D8Rat58 | B | B | H | B | B | B | B | H | H | B | H | B | H | B | B | H | H | B | H |
| D8Rat56 | B | B | H | B | B | B | B | H | H | B | B | B | B | H | H | H | H | B | H |
| D8Rat68 | H | B | H | B | B | B | B | B | B | B | B | B | B | H | H | H | B | B | H |
| D8Mit6 | B | B | H | B | B | B | *U* | H | H | B | H | B | B | H | H | H | B | B | H |
| D8Mgh9 | H | H | *U* | H | B | B | *U* | B | *U* | H | H | B | B | H | H | H | B | H | B |
| D8Rat150 | H | B | B | B | B | B | B | B | H | H | H | H | H | H | H | H | B | H | B |
| D8Rat21 | B | B | B | B | B | B | B | B | B | H | H | H | H | H | H | B | B | H | H |
| D8Rat130 | B | *U* | B | B | B | B | B | B | B | B | B | H | B | H | H | B | B | H | H |
| D8Rat123 | B | B | B | H | B | H | B | B | H | B | B | H | B | H | H | B | B | H | H |
| D8Rat202 | B | H | B | H | B | H | B | B | H | H | B | H | B | H | H | B | B | H | H |
| D8Rat7 | B | H | B | H | B | B | H | B | B | B | B | H | B | H | H | B | B | H | B |

| **Chr. 9** | **1H** | **4H** | **7H** | **25H** | **27H** | **6B** | **O8B** | **11B** | **13B** | **2H** | **3H** | **10H** | **15H** | **17H** | **18H** | **23H** | **29H** | **31H** | **10B** |
|---|---|---|---|---|---|---|---|---|---|---|---|---|---|---|---|---|---|---|---|
| D9Rat88 | H | H | B | H | B | H | B | B | H | H | H | H | H | H | B | B | H | B | H |
| D9Rat131 | B | B | B | H | B | H | B | B | H | H | H | H | H | H | H | B | H | B | H |
| D9Rat158 | H | B | B | H | B | B | H | H | H | B | H | H | H | H | B | B | H | B | H |
| D9Rat180 | H | B | B | H | B | B | H | B | H | B | H | H | H | H | B | B | H | B | H |
| D9Rat104 | B | B | B | H | H | B | H | H | H | H | H | H | H | H | H | B | H | B | B |
| D9Rat60 | B | B | B | H | B | H | B | H | B | H | H | B | H | H | B | B | B | B | B |
| D9Rat19 | B | B | B | H | B | H | B | H | B | H | B | H | B | H | B | B | B | H | B |
| D9Rat12 | B | B | B | H | H | B | B | H | B | B | B | H | B | B | B | B | B | H | H |
| D9Rat171 | B | H | B | H | H | B | B | H | B | B | B | H | B | H | B | H | B | H | H |
| D9Rat4 | B | B | B | H | H | B | B | B | H | B | B | H | B | H | B | H | B | B | H |
| D9Rat153 | B | B | B | H | H | B | B | B | H | H | H | H | H | H | H | H | B | H | B |
| D9Rat108 | H | H | H | H | H | B | B | B | H | H | H | H | B | H | H | H | B | H | B |
| D9Rat1 | H | H | H | H | B | B | B | B | H | H | B | H | B | B | B | H | B | H | H |

| **Chr. 10** | **1H** | **4H** | **7H** | **25H** | **27H** | **6B** | **O8B** | **11B** | **13B** | **2H** | **3H** | **10H** | **15H** | **17H** | **18H** | **23H** | **29H** | **31H** | **10B** |
|---|---|---|---|---|---|---|---|---|---|---|---|---|---|---|---|---|---|---|---|
| D10Rat95 | H | H | H | B | B | B | B | H | B | B | H | H | B | H | H | H | H | H | B |
| D10Rat218 | H | H | H | B | B | B | B | H | B | B | B | B | B | H | H | H | H | H | B |
| D10Rat121 | H | H | H | B | B | H | B | B | H | B | H | B | H | H | H | H | H | H | H |
| D10Rat45 | B | H | H | B | B | H | B | B | B | B | B | B | B | H | H | H | H | H | H |
| D10Rat71 | B | H | H | H | B | B | B | B | H | B | B | B | B | B | H | H | H | B | B |
| D10Mit4 | B | H | H | H | H | B | *U* | B | B | B | B | B | B | B | B | H | H | H | B |
| D10Rat166 | B | H | H | H | H | H | H | B | B | B | B | B | B | B | B | B | H | B | B |
| **D10Rat102** | **B** | **H** | **H** | **H** | **H** | **H** | **H** | **H** | **H** | **B** | **B** | **B** | **H** | **B** | **B** | **B** | **B** | **B** | **B** |
| D10Rat26 | B | H | H | H | H | B | H | H | H | B | H | B | B | B | B | B | H | B | B |
| D10Mit7 | H | H | B | H | H | H | H | H | H | B | H | B | B | B | B | H | B | B | B |
| D10Rat267 | H | H | B | B | H | H | H | H | H | H | H | B | H | B | B | H | B | B | H |
| D10Rat228 | H | B | B | B | H | H | H | H | H | H | B | B | B | B | H | H | B | B | H |
| D10Rat7 | H | B | H | B | H | H | H | B | H | H | H | H | H | B | H | H | H | B | H |
| D10Rat226 | H | B | H | H | H | H | H | B | H | H | H | H | H | H | H | H | H | B | B |

| **Chr. 11** | **1H** | **4H** | **7H** | **25H** | **27H** | **6B** | **O8B** | **11B** | **13B** | **2H** | **3H** | **10H** | **15H** | **17H** | **18H** | **23H** | **29H** | **31H** | **10B** |
|---|---|---|---|---|---|---|---|---|---|---|---|---|---|---|---|---|---|---|---|
| D11Rat28 | H | H | H | B | B | B | B | H | B | H | H | B | H | B | B | B | H | H | H |
| D11Rat20 | H | B | H | B | B | B | B | H | B | H | H | B | H | B | B | B | H | H | H |
| D11Rat16 | H | H | B | B | B | B | B | B | B | H | H | H | H | B | B | B | H | H | H |
| D11Rat7 | H | B | B | B | B | B | B | B | H | B | H | H | H | B | B | B | H | H | B |
| D11Rat65 | H | B | B | B | B | B | B | B | H | B | B | H | B | B | H | B | B | B | B |
| D11Rat94 | B | H | B | B | B | H | B | H | H | B | B | H | B | B | B | B | B | B | H |
| D11Rat47 | B | H | B | H | B | H | B | H | H | B | H | H | B | B | B | B | B | B | B |
| D11Rat1 | H | H | B | B | B | H | B | H | H | H | B | B | B | B | B | H | B | H | H |

| **Chr. 12** | **1H** | **4H** | **7H** | **25H** | **27H** | **6B** | **O8B** | **11B** | **13B** | **2H** | **3H** | **10H** | **15H** | **17H** | **18H** | **23H** | **29H** | **31H** | **10B** |
|---|---|---|---|---|---|---|---|---|---|---|---|---|---|---|---|---|---|---|---|
| D12Rat56 | B | H | B | B | H | H | H | H | B | H | B | B | B | H | B | H | H | B | H |
| D12Rat40 | B | H | B | H | H | H | H | H | B | H | B | B | B | H | H | H | H | B | H |
| D12Rat61 | B | H | B | H | B | H | H | H | B | H | H | B | B | H | H | H | H | B | H |
| D12Rat10 | B | H | B | H | B | H | H | H | H | H | B | B | B | B | B | H | H | B | H |
| D12Rat42 | H | B | B | H | B | H | H | H | H | H | H | B | B | B | B | H | H | B | H |
| D12Rat14 | H | H | B | H | B | H | B | H | H | H | H | B | B | B | B | H | H | B | H |
| D12Rat16 | B | H | B | H | B | H | B | H | H | H | B | B | B | B | B | H | H | B | H |
| D12Rat36 | H | B | B | B | B | H | B | H | H | H | H | B | B | B | B | H | H | B | H |

| MARKERS | LT-Resistant | | | | | | | | | LT-Sensitive | | | | | | | | | |
|---|---|---|---|---|---|---|---|---|---|---|---|---|---|---|---|---|---|---|---|
| **Chr. 13** | **1H** | **4H** | **7H** | **25H** | **27H** | **6B** | **O8B** | **11B** | **13B** | **2H** | **3H** | **10H** | **15H** | **17H** | **18H** | **23H** | **29H** | **31H** | **10B** |
| D13Mit1 | *U* | H | H | H | H | B | *U* | H | H | B | H | H | H | B | H | B | H | H | B |
| D13Rat113 | H | H | B | B | B | B | B | H | H | H | H | H | H | H | B | H | H | B | B |
| D13Rat88 | H | H | B | H | B | B | H | H | H | H | H | H | H | H | H | H | H | H | B |
| D13Rat126 | H | H | H | B | B | B | H | H | H | H | H | H | H | H | B | H | H | H | B |
| D13Rat131 | B | H | H | B | B | B | H | H | B | H | H | H | H | H | B | B | H | B | B |
| D13Rat152 | B | H | B | B | B | B | H | H | H | H | H | H | H | H | H | B | B | B | B |

| **Chr. 14** | **1H** | **4H** | **7H** | **25H** | **27H** | **6B** | **O8B** | **11B** | **13B** | **2H** | **3H** | **10H** | **15H** | **17H** | **18H** | **23H** | **29H** | **31H** | **10B** |
|---|---|---|---|---|---|---|---|---|---|---|---|---|---|---|---|---|---|---|---|
| D14Rat77 | H | H | B | B | B | B | H | B | B | H | H | B | H | B | B | H | B | B | H |
| D14Rat8 | B | H | B | H | B | B | B | B | H | H | H | B | H | B | B | H | B | H | H |
| D14Rat64 | B | H | H | H | B | B | H | H | H | B | H | B | H | B | B | H | B | H | H |
| D14Rat37 | B | H | H | B | B | B | H | H | H | B | H | H | H | B | B | B | B | H | H |
| D14Rat94 | B | H | H | B | B | H | H | B | H | B | H | H | H | B | B | B | B | H | B |
| D14Rat38 | B | H | H | B | B | H | H | B | B | H | H | H | H | H | B | H | B | H | B |

| **Chr. 15** | **1H** | **4H** | **7H** | **25H** | **27H** | **6B** | **O8B** | **11B** | **13B** | **2H** | **3H** | **10H** | **15H** | **17H** | **18H** | **23H** | **29H** | **31H** | **10B** |
|---|---|---|---|---|---|---|---|---|---|---|---|---|---|---|---|---|---|---|---|
| D15Rat1 | H | H | B | H | B | B | B | H | H | B | B | H | B | B | B | B | H | B | B |
| D15Mit3 | B | H | B | H | B | B | *U* | B | B | B | B | B | B | B | B | B | B | B | B |
| D15Rat6 | H | H | B | B | B | B | B | B | H | B | B | B | B | B | B | H | B | B | B |
| D15Rat123 | H | B | B | B | H | B | B | B | B | B | H | B | H | H | B | B | B | B | B |
| D15Rat68 | H | H | H | B | H | B | B | B | B | B | H | B | B | B | B | B | B | B | B |
| D15Rat21 | H | H | B | B | H | B | B | B | H | H | H | H | B | B | H | B | B | B | B |
| D15Rat101 | B | H | B | H | H | B | B | H | H | H | B | H | B | B | H | H | B | B | B |
| D15Rat107 | B | H | B | H | B | B | B | B | H | B | B | B | B | B | H | H | H | B | B |

| **Chr. 16** | **1H** | **4H** | **7H** | **25H** | **27H** | **6B** | **O8B** | **11B** | **13B** | **2H** | **3H** | **10H** | **15H** | **17H** | **18H** | **23H** | **29H** | **31H** | **10B** |
|---|---|---|---|---|---|---|---|---|---|---|---|---|---|---|---|---|---|---|---|
| D16Mit2 | B | B | H | B | B | B | B | B | H | H | H | H | H | H | H | B | B | H | B |
| D16Mit5 | H | B | H | B | B | B | H | B | H | H | H | B | H | H | H | B | B | H | B |
| D16Rat41 | B | B | H | B | B | B | *U* | B | *U* | H | H | H | H | H | H | B | B | H | B |
| D16Rat67 | H | H | B | B | B | B | H | B | H | H | H | H | H | H | H | H | B | B | B |
| D16Rat72 | H | H | H | H | B | B | H | B | H | B | H | H | B | H | B | H | H | B | H |
| D16Rat66 | H | H | H | H | B | B | *U* | B | *U* | B | H | H | H | H | B | H | H | B | *U* |
| D16Rat53 | H | H | H | H | B | B | B | B | H | B | H | H | H | H | B | H | H | B | H |
| D16Rat34 | B | H | H | H | H | B | B | H | H | H | H | H | H | H | B | H | B | B | H |
| D16Rat14 | B | H | B | H | H | B | *U* | H | H | H | H | B | H | H | H | H | B | B | H |
| D16Rat15 | B | H | B | H | H | B | B | H | H | B | B | H | B | H | H | H | B | B | H |

| **Chr. 17** | **1H** | **4H** | **7H** | **25H** | **27H** | **6B** | **O8B** | **11B** | **13B** | **2H** | **3H** | **10H** | **15H** | **17H** | **18H** | **23H** | **29H** | **31H** | **10B** |
|---|---|---|---|---|---|---|---|---|---|---|---|---|---|---|---|---|---|---|---|
| D17Rat11 | B | H | H | B | H | B | H | B | B | H | B | B | H | B | B | H | H | H | H |
| D17Rat144 | B | H | B | B | B | B | B | B | B | H | B | B | B | B | B | H | H | H | B |
| D17Mit3 | B | B | B | B | B | B | *U* | B | B | H | B | B | B | B | B | B | *U* | B | H |
| D17Rat151 | H | H | B | B | H | B | B | B | B | H | B | B | H | B | B | B | H | B | B |
| D17Mit6 | B | H | B | B | H | B | *U* | B | B | *U* | B | B | H | H | H | H | B | B | B |
| D17Rat50 | B | H | H | B | H | B | B | H | B | H | B | H | B | H | H | H | B | B | B |

| **Chr. 18** | **1H** | **4H** | **7H** | **25H** | **27H** | **6B** | **O8B** | **11B** | **13B** | **2H** | **3H** | **10H** | **15H** | **17H** | **18H** | **23H** | **29H** | **31H** | **10B** |
|---|---|---|---|---|---|---|---|---|---|---|---|---|---|---|---|---|---|---|---|
| D18Rat112 | H | H | B | H | H | H | H | H | B | H | H | H | H | H | H | B | B | H | B |
| D18Rat29 | B | H | B | H | B | H | H | H | B | B | H | H | H | H | H | B | B | B | B |
| D18Rat47 | B | H | H | H | H | H | H | H | B | B | H | B | H | H | H | B | B | H | B |
| D18Rat103 | B | H | H | H | H | H | H | H | B | B | B | H | B | H | H | B | B | H | B |
| D18Rat99 | B | H | H | H | B | H | H | H | B | B | B | H | B | H | H | H | B | B | B |
| D18Rat55 | B | H | H | H | H | H | B | H | H | B | B | H | B | H | B | H | H | B | B |
| D18Rat89 | B | H | H | H | H | H | H | B | H | B | B | H | B | H | B | H | H | B | B |
| D18Rat9 | B | B | H | H | H | H | H | B | H | H | B | H | B | H | B | H | B | H | B |
| D18Rat5 | H | B | H | H | H | B | H | B | H | B | B | B | B | H | B | H | B | H | B |
| D18Mit9 | H | B | H | H | H | B | *U* | B | H | B | H | B | B | H | B | H | B | H | B |

| **Chr. 19** | **1H** | **4H** | **7H** | **25H** | **27H** | **6B** | **O8B** | **11B** | **13B** | **2H** | **3H** | **10H** | **15H** | **17H** | **18H** | **23H** | **29H** | **31H** | **10B** |
|---|---|---|---|---|---|---|---|---|---|---|---|---|---|---|---|---|---|---|---|
| D19Rat19 | H | H | H | H | H | H | *U* | H | *U* | H | H | B | B | H | *U* | B | B | B | B |
| D19Rat56 | H | H | B | H | H | H | H | H | B | H | H | B | H | H | B | B | B | B | B |
| D19Rat48 | H | H | H | B | H | H | H | B | B | H | B | H | B | H | B | B | B | H | B |
| D19Mit7 | H | H | H | B | H | B | *U* | H | B | H | H | H | B | H | B | H | B | B | B |
| D19Rat71 | H | B | H | B | B | B | B | H | B | H | H | H | B | H | B | H | B | B | B |
| D19Rat64 | H | H | B | B | B | B | H | H | B | H | H | H | B | H | B | H | B | H | B |
| D19Rat61 | H | H | H | B | B | B | H | H | B | B | H | H | B | H | B | H | H | H | B |
| D19Rat5 | *U* | B | H | B | B | B | *U* | B | B | H | H | B | H | H | H | H | H | H | B |

| **Chr. 20** | **1H** | **4H** | **7H** | **25H** | **27H** | **6B** | **O8B** | **11B** | **13B** | **2H** | **3H** | **10H** | **15H** | **17H** | **18H** | **23H** | **29H** | **31H** | **10B** |
|---|---|---|---|---|---|---|---|---|---|---|---|---|---|---|---|---|---|---|---|
| D20Rat41 | B | B | B | B | B | H | B | H | B | H | H | B | H | H | B | B | B | H | B |
| D20Mgh5 | B | B | B | B | B | H | B | H | B | B | H | B | H | H | B | B | B | H | H |
| D20Rat4 | B | H | B | B | B | B | B | H | H | H | H | B | H | B | H | B | B | H | H |
| D20Rat75 | H | H | B | H | H | H | B | H | H | H | H | B | H | H | *U* | B | B | B | H |
| D20Rat23 | H | H | B | H | H | H | B | B | B | B | H | B | H | H | H | B | B | B | B |
| D20Rat9 | B | H | B | H | H | H | B | B | B | B | B | B | H | H | H | H | B | H | B |
| D20Rat10 | B | H | B | B | H | B | B | B | B | B | B | B | H | H | H | H | B | H | B |
| D20Rat55 | B | H | B | B | H | H | B | B | B | H | B | B | H | B | H | B | H | H | B |
| D20Mit1 | B | H | B | H | H | H | B | B | B | B | B | B | B | B | H | B | B | H | B |
